# Achieving effective interlayer bonding of PLA parts during the material extrusion process with enhanced mechanical properties

**DOI:** 10.1038/s41598-023-33510-7

**Published:** 2023-04-26

**Authors:** M. Saravana Kumar, Muhammad Umar Farooq, Nimel Sworna Ross, Che-Hua Yang, V. Kavimani, Adeolu A. Adediran

**Affiliations:** 1 Graduate Institute of Manufacturing Technology, National Taipei University of Technology, Taipei, 10608 Taiwan; 2 School of Mechanical Engineering, University of Leeds, Leeds, LS2 9JT UK; 3 Department of Mechanical Engineering, Saveetha School of Engineering, SIMATS, Chennai, Tamilnadu India; 4 Department of Mechanical Engineering, Karpagam Academy of Higher Education, Coimbatore, Tamil Nadu 641021 India; 5 Department of Mechanical Engineering, Landmark University, P.M.B. 1001, Omu-Aran, Kwara State Nigeria; 6 Department of Mechanical Engineering Science, University of Johannesburg, Johannesburg, South Africa

**Keywords:** Mechanical engineering, Materials science

## Abstract

The additive manufacturing technique of material extrusion has challenge of excessive process defects and not achieving the desired mechanical properties. The industry is trying to develop certification to better control variations in mechanical attributes. The current study is a progress towards understanding the evolution of processing defects and the correlation of mechanical behavior with the process parameters. Modeling of the 3D printing process parameters such as layer thickness, printing speed, and printing temperature is carried out through L27 orthogonal array using Taguchi approach. In addition, CRITIC embedded WASPAS is adopted to optimize the parts' mechanical attributes and overcome the defects. Flexural and tensile poly-lactic acid specimens are printed according to ASTM standards D790 and D638, respectively, and thoroughly analyzed based on the surface morphological analysis to characterize defects. The parametric significance analysis is carried out to explore process science where the layer thickness, print speed, and temperature significantly control the quality and strength of the parts. Mathematical optimization results based on composite desirability show that layer thickness of 0.1 mm, printing speed of 60 mm/s, and printing temperature of 200 °C produce significantly desirable results. The validation experiments yielded the maximum flexural strength of 78.52 MPa, the maximum ultimate tensile strength of 45.52 MPa, and maximum impact strength of 6.21 kJ/m^2^. It is established that multiple fused layers restricted the propagation of cracks with minimum thickness due to enhanced diffusion between the layers.

## Introduction

In recent years, additive manufacturing technology has been used to manufacture a variety of aeronautical and healthcare applications^1^. Various additive manufacturing procedures exist as referred by the ISO/ASTM 52,900:2015 standard, with material extrusion process (MEX) being one of the most extensively utilized owing to its flexibility, minimal wastage of materials, and ability to print different geometry with structural support^2,3^. Poly-Lactic Acid (PLA), Nylon, Acrylonitrile Butadiene Styrene (ABS), and Polyethylene Terephthalate Glycol (PETG) were the filament materials used in MEX-process. Since it is a biodegradable polymer ideal for tissue/bone engineering disciplines, thermoplastic PLA is a regularly used 3D printing material^4^. The performance of the printed parts was affected by some common defects such as overheating, layer shifting, under and over extrusion, gaps in top layer, curing and rough corners^5^. Hence, the effort to overcome the defects accelerated over the past few years. However, to maintain the consistency of the properties of the MEX parts, the printing parameters needed to be optimized to enhance the mechanical properties of the printed parts. Some research was conducted to analyze the physical behavior of 3D printed objects to expand the engineering use of 3D printing technology. Liu H et al. found that the composite filament printed in the parallel direction has significantly better tensile performance than the composite filament printed in the vertical direction^6^. The support structure was one of the most significant constraints in constructing 3D printed parts and also best print support structure dependent on the orientation.

Multi-objective optimization was used to reduce the quantity of materials needed for the structural support, lowering material costs, and increasing work efficiency^7^. The reuse of 3D printed items also contributes significantly to decreasing environmental issues. Even though regular PLA has good characteristics, recycled PLA has a lower viscosity, higher crystallinity, and less transparency^8^. The researchers found that the tensile qualities of 3D printed PLA material increase with increasing infill density and that concrete patterns had better tensile properties than grid and tri hexagon patterns^9^. The rise in tensile strength and young's modulus was attributed to an increase in infill density in numerous studies, and the anisotropy to the tension of 3D printed materials was demonstrated by shear tests, shear modulus, and shear rate^10^. Thermal stability analysis was used to assess the impact of printing temperature on 3D printed PLA material, and several experiments were carried out by adding clay nanoparticles to the PLA material to boost thermal stability^11^. A few researchers looked into the impact of infill density on the heat-resistant carbon fiber reinforced PETG material and found that a 60 percent infill density combined with annealing effects resulted in good wear resistance^12^. The influence of layer thickness was also documented in certain studies, in which the printing layer thickness was found to impact surface roughness and print orientation significantly. According to the findings, edge-oriented 3D printed PLA components with lower layer thickness and flat-oriented 3D printed PLA parts with higher layer thickness have better bonding strength^13^. Abdalla A et al. revealed that the vertically oriented materials have excellent resistivity and electron transport kinetics^14^. The infill pattern influenced the tensile characteristics of the manufactured 3D printed PLA materials. For 3D printing, various infill patterns such as grid, zig-zag, and concrete patterns were used, with zig-zag machining demonstrating improved tensile performance^15^. The effects of modifying the orientation and infill density on the tensile characteristics of 3D printed PLA and HT-PLA materials were studied. The HT-PLA with X orientation and 100 percent infill density substantially impact the tensile performance^16^. A comprehensive list of studies which incorporate a range of process parameters and their effects are tabulated in Table 1.

**Table 1:** A comprehensive overview of MEX input parameters and their effect on different responses as reported in literature.

Sr. no.	Source	Year	Material and 3D printer	Parameters and conditions	Conclusion
1	Abbas et al.^17^	2017	Ultimaker and Material Extrusion (MEX) with PLA material	Layer thickness: 0.1 mm Print speed: 75 mm/s Part orientation: 90° Shell thickness: 1.6 mm Infill densities: 80%, 65%, 50%, 35% and 20% Responses: Compression test ASTM D695 standard	High mechanical resistance was achieved through 80% infill density High compression was resulted by solid specimens. However, reducing printing time could be achieved through lower infill density
2	Mishra et al.^18^	2021	Ultimaker and PLA material	Plate temperature: 60 °C Print temperature: 215 °C Layer thickness: 0.2 mm Infill densities: 7 types and 12 patterns Responses: ASTM D256 Izod Impact test	Superior energy absorbing potential was observed with 85% infill density
3	Hussin et al.^19^	2020	QiDi Tech 3D with ABS material	Print speed: 60 mm/s Plate temperature: 80 °C Print temperature: 230 °C Layer thickness: 0.2 mm Infill densities: 100% with triangular and line pattern Responses: Tensile test rate ASTM D638 Compression Test ASTM D695	High mechanical stress was observed through high print speed. Infill pattern directly affect mechanical properties with the print pattern. Authors recommended to quantify the effect of print speed and layer thickness
4	Zaldivar et al.^20^	2017	Stratasys 400 ULTEM® Material Extrusion 9085 Material	Layer thickness: 0.254 mm Infill orientation: 0°, 90° Responses: ASTM D638-03 Tensile test	Highest tensile strength was observed at Edge 0° along with highest Modulus
5	Yu et al.^21^	2019	Material Extrusion of PLA material	Print speed: 30, 40, 50 mm/s Infill densities: 40, 80, 100% Layer thickness: 0.15, 0.2, 0.25 mm Responses: Tensile test GB/T 1040.2–2006 Compression test GB/T 1041–1992	Mechanical properties decreased with the increase in layer thickness Tensile strength was significantly influenced by layer thickness among all other variables
6	Wang et al.^22^	2020	Markforged X7 Material Extrusion of Polyamide composite	Print speed: 0.4, 4, 40 mm/min Layer thickness: 0.2 mm Print temperature: 275 °C Responses: Tensile strength Strain rate	Improved stiffness was observed with higher infill densities which also improved tensile properties. The print speed is a vital parameter affecting mechanical properties
7	Dpu et al.^23^	2020	3D RepRap Kossel with PLA material	Infill density: 100% Plate temperature: 50 °C Print speed: 50 to 400 mm/min Print temperature: 190 to 230 °C Layer thickness: 0.2 to 0.4 mm Layer width: 0.86 to 1.5 mm Responses: Tensile test ASTM D3039-07	Increasing layer thickness decreased tensile strength, however, an increasing trend was observed with enhancing print temperature
8	Kechagias et al.^24^	2022	Craftbot® Plus 3D printer for fused filament fabrication of PLA/Coconut wood compound	Layer thickness: 0.1, 0.3 mm Nozzle temperature: 180 to 220 °C Deposition angle: 0, 45, 90° Print speed: 30, 40, 50 mm/s Responses: ultimate tensile strength and elasticity module	The layer thickness is found as a significant parameter but dependent on printing speed for interlaminar bonding conditions
9	Fountas et al.^25^	2022	Craftbot® plus Pro 3D printer for FET-G specimens	Layer thickness: 0.1, 0.2, 0.3 Infill density: 80, 90, 100% Print speed: 40, 50, 60 mm/s Print temperature: 230, 240, 250° Responses: Flexural stress	Layer thickness affected bonding formation and diffusion because of thermal energy absorption among passes
10	Silva et al.^26^	2021	PolyJet™, model EDEN 260 V by Stratasys for printing of gyroid-based structures	Bed temp: 60° Infill density: 20, 50, 80, 100% Layer height: 0.2 mm Responses: Tensile test ISO 527/1 (1996) 527/2 (1996) Impact test	The infill density of 100% with a raster angle of 0° produced superior mechanical results
11	Butt et al.^27^	2022	Anet® ET4 Pro (Shenzen Anet Technology Company, Hong Kong) 3D printer for printing PLA and ABS	Layer thickness: 0.1, 0.2, 0.3, 0.4 mm Infill density: 100% Print speed: 50 mm/s Responses: Surface texture, hardness and tensile strength	PLA showed superior hardness results as compared to others. Similar fracture load values were observed for PLA and ABS samples

Sachini Wickramasinghe et al. investigated the functional properties of the fiber reinforced composite fabricated through MEX and also examined their defects based on the infill density, print orientation and layer thickness. They have found that some common defects, such as void formation and surface roughness, were resolved by optimizing those process parameters^12^. Similarly, Yuhan Liao et al. analyzed the interrelationship between crystallinity and porosity, which influences the mechanical properties of the printed parts. Further, they found that the bed temperature and print orientation considerably affect the printed parts' microstructure^28^. Dawoud et al. examined the scaffolding angle and raster gap to enhance the mechanical properties of the printed parts. Further, the ABS filament was infused with graphite flakes to improve the tribological properties. They  reported that a 90° scaffold angle with a negative gap has enhanced mechanical and tribological properties^29^. Gianluca Cicala et al. investigated printing the complex shape with high accuracy. Thermogravimetric analyses were performed on the printed parts to examine their rheology and carried out the tensile test to validate their experiments^4^. Patil et al.^30^ optimized material extrusion process parameters for roughness, printing time, and length of filament consumed. The layer thickness of 0.2 mm and printing speed of 100 mm/h were found to be the most optimal selection for achieving desired results. Luzanin et al.^31^ evaluated tensile strength (found optimized at 0.2 mm) and mesostructure in MEX-fabricated polylactic acid. It was found that layer thickness and its quadratic effect affected the tensile strength significantly. Hsueh et al.^32^ studied mechanical properties of 3D-Printed PLA against ASTM D638, ASTM D3410 and ASTM D790 standards. It was found that with the increase in print temperature or speed, the mechanical properties of the printed PLA material increased. Likewise, Rodríguez-Panes et al.^33^ evaluated tensile mechanical behavior of printed PLA and resulted that the increased layer height (0.1–0.2 mm) caused the tensile strength to reduce by 11%. On the other hand, Wang et al.^34^ compared injection molded PLA with 3D-printed PLA and resulted 114% higher performance of MEX-based PLA in Izod impact strength which certainly outperformed in quality matrix.

The literature survey showed that the input printing parameters influenced the mechanical behavior of the 3D-printed parts. Multi-criteria decision-making approach help to improve the manufacturing process to obtained unique solution in manufacturing industries. Conversely these approaches are rarely reported for 3D printing process. Effective utilization of these approaches is believed to be better solution in solving decision making problem in materials printing. Therefore, this research is going to focus on determination of effective control over the process that ultimately improving the mechanical attributes. In addition, predictive models are presented to characterize the influence of process parameters on the response indicators. A comprehensive study on the mechanical attributes of the material extrusion processed parts is required where the instant predictive model validated through experiments provides aid in manufacturing planning. In this regard, this research aims to optimize the important 3D printing process parameters to improve mechanical performance. In most of the literature, they have chosen a wide range of printing process parameters which was difficult to find an exact optimal parameter setting for the improved mechanical performance of PLA parts. So, the input parameters were chosen in a close range which attributed to the attainment of precise optimal parameter setting with enhanced interlayer bonding of PLA parts. Especially the layer thickness settings have a huge impact on attaining effective interlayer bonding. Hence, the close range of input parameter settings such as layer thickness (0.1, 0.15, and 0.2 mm), printing temperature (195, 200, and 205 °C), printing speed (50, 60, and 70 mm/s) with 100 percent infill density and bed temperature of 70 °C were used in this study to create the PLA parts. Mechanical testing such as tensile, and flexural was performed for all the combinations. SEM analysis was also used to analyze the fractured surface of the printed PLA specimens. These fractures displays the effective and poor interlayer bonding of parts. Further, the Taguchi with Criteria Importance through Intercriteria Correlation (CRITIC) embedded Weighted Aggregated Sum Product Assessment (WASPAS) based multi-criteria decision-making approach is carried out to assess the percentage contribution of MEX input factors on the printed PLA's flexural strength and ultimate tensile strength. Additionally, the mathematical model predicts the values of output responses such as flexural strength and ultimate tensile strength under the optimum layer thickness, printing speed, and printing temperature with minimum base runs.

## Materials and methods

## Materials

PLA is a thermoplastic polyester made from natural and renewable resources such as corn, cassava, and sugarcane^35^. A premium grade PLA (polylactic acid) filament was purchased from Tesseract, Mumbai, Maharashtra, India and it was used to print the specimens with different layer thicknesses, printing speeds, and temperatures.

## Material extrusion (MEX) and the parameters

Material Extrusion (MEX) is defined by the ISO/ASTM 52,900:2015 standard as the “additive manufacturing process in which material is selectively dispensed through a nozzle or orifice”. SolidWorks 2019 was used to create all of the test specimen models. Ultimaker Cura 15.04.3 software was used for the slicing of the modelled specimens. The designed parts were converted to .STL files with reference to ISO/ASTM 52,900 standard. The Ultimaker Cura 15.04.3 software requires STL files to be imported. In this research, layer thickness of 0.1, 0.15, and 0.2 mm, printing temperature of 195, 200, and 205 °C, and printing speed of 50, 60, and 70 mm/s were chosen based on the manufacturer’s suggestion and the research gap. Most of the parameter settings were taken in a wide range which makes it more difficult to identify the precise process parameters. So, in this research, the process parameters were chosen in a close range in order to find out the exact optimal setting which results in improved mechanical properties. In the literature survey, it was reported that the reduced layer thickness exhibits better performance. So, in the research, all three layer thickness was chosen in a close range from 0.1 to 0.2 mm. These parameters were adopted to print the specimens (as shown in Table 2) and also a printing angle of 90°^13^ and 100% infill percentage were followed to print the PLA specimens in a set of experiments. These fixed parameters were taken from the literature where they have already proved that adopting those parameters shown improved performance when compared with the others parameters. Some of the already well know proven parameters settings are 100% infill density. Similarly, the Zig-Zag pattern was considered for this research based on the reference Yeoh et al.^15^. They have proved that PLA with zig-zag infill pattern has higher tensile strength of 23.409 MPa compared to PLA with grid and concentric infill pattern. These selected parameters are given as an input to the software. The diameter of the PLA filament was 2.85 mm, and it was extruded in the nozzle having 0.4 mm in diameter. The vertical (90°) building orientation with respect to ISO/ASTM 52,900 standard was assigned for printing tensile, impact and flexural specimen. Moreover, this orientation does not require support structure in reference to ISO/ASTM 52,900 standard. The G-code will be generated, and the generated G-code will be sent to the 3D printer as an input. The layer-by-layer printing operations was carried out in the 3D printer based on the supplied process parameters and G-code.

**Table 2:** Investigated and fixed parameters.

Selected parameters	Levels			Fixed parameters	
	Low	Middle	High		
Layer thickness (mm)	0.1	0.15	0.2	Infill pattern	Zig-Zag pattern (based on recommendations by Yeoh et al. ^15^ where authors compared multiple patterns against mechanical properties)
Printing speed (mm/s)	50	60	70	Infill density (%)	100 (based on recommendations by Kechagias et al. ^24^. Also Fountas et al. ^25^ found superior flexural strength)
Printing temperature (°C)	195	200	205	Bed temperature (°C)	60 (based on recommendations by Silva et al. ^26^)

**Figure 1:**
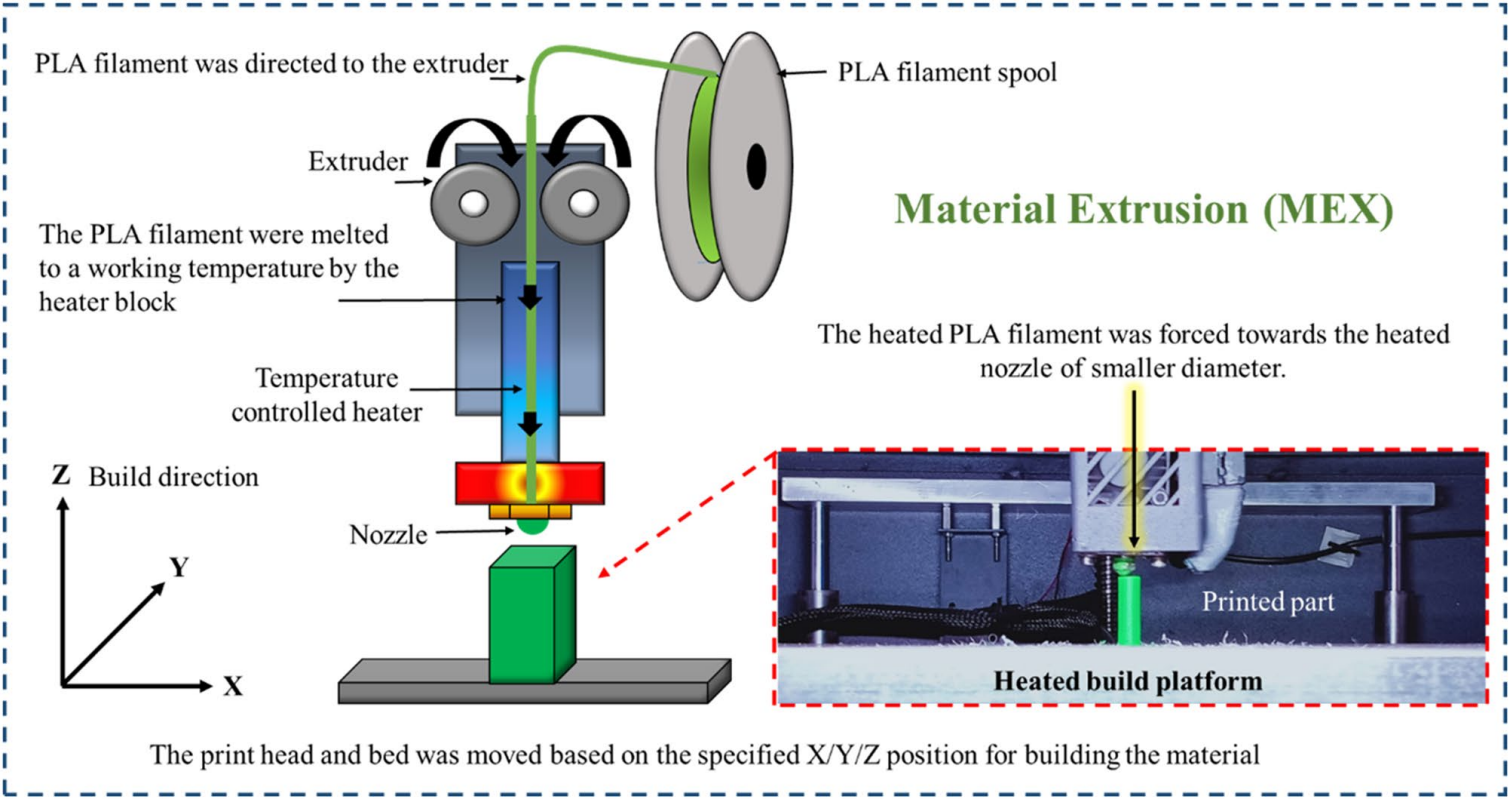
Schematic representation of Material Extrusion (MEX) process with the printed PLA specimen.

A Drona C300 3D printer was employed to print the required specimens, as shown in Fig. 1. Even though, it was a well-known machine, this schematic representation helps the budding researcher to understand more about the extrusion mechanism. In the extruder setup, the PLA wire filaments are inserted. The melted PLA filament was extruded from the nozzle and started printing on the preheated bed. 60 °C bed temperature must be maintained in order to stick the specimen in the bed and also helps to start to print in the exact place of the starting point.

**Figure 2:**
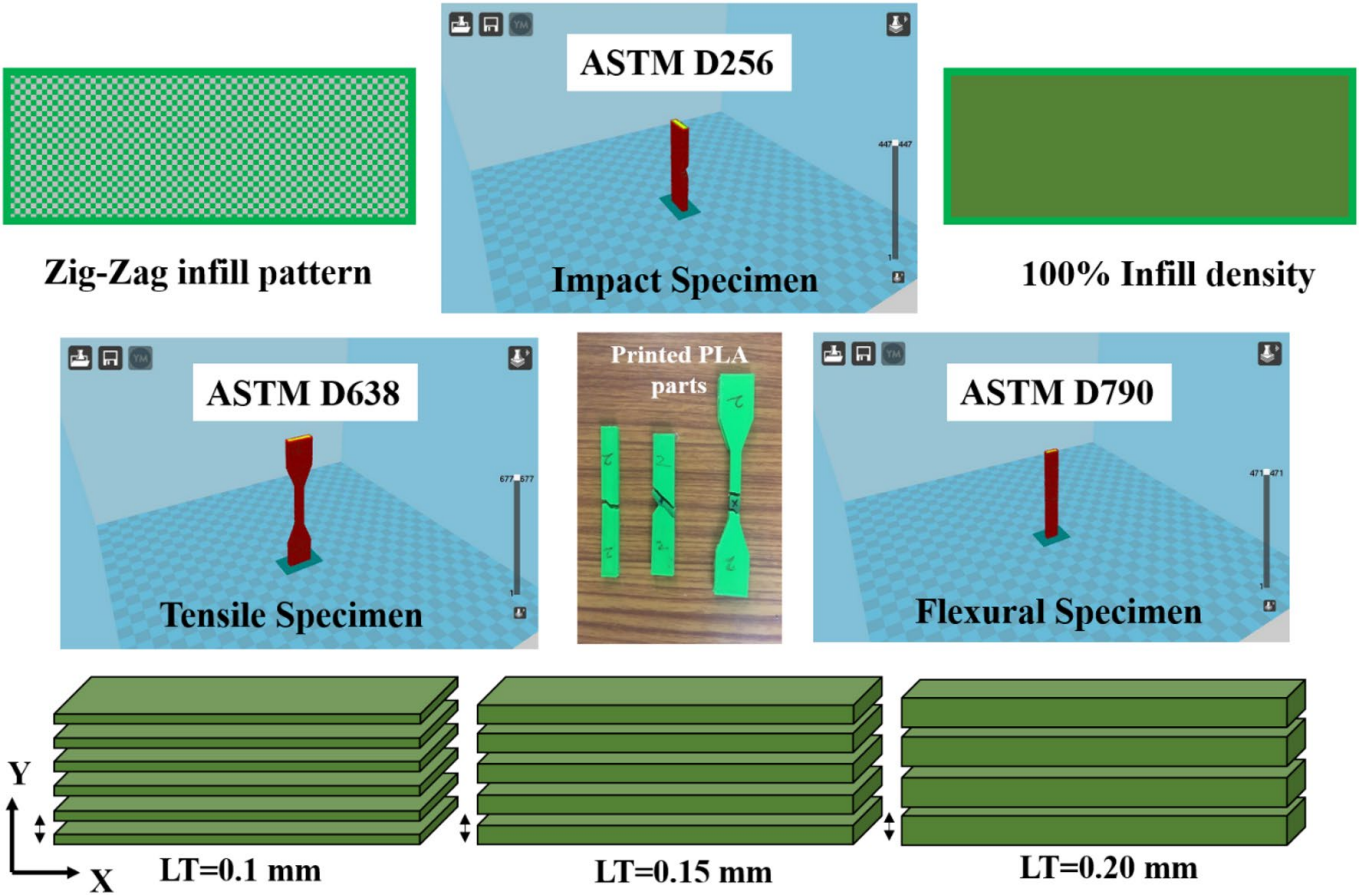
Schematic representation of Layer thickness, zig-zag infill pattern, 100% infill density and slicing software images of the tensile, impact and flexural specimens with printed PLA parts.

Similarly, Zig-Zag pattern with 100% infill density was adapted to the print the specimens which was chosen based on the literature^15^. Layer-by-layer printing with reference to ISO/ASTM 52,900 standard was done according to the slicing instructions which was shown in the Fig. 2. All test specimens were printed with various layer thicknesses, printing speeds, and temperatures.

## Experimental design

In the full factorial design, a wide range of parametric settings are tested which significantly increase cost and time of the project. Therefore, different robust design of experiment approaches are used such as Taguchi or response surface methodology and Taguchi which offer higher confidence levels of experimental campaign, assist in modelling and directly reduce time and cost. Box-Behnken design (BBD) and Taguchi are one of the widely used fractional factorial experimental approaches which comes under response surface methodology. Kechagias and Vidakis established supremacy of BBD and compared its benefits with full factorial design during fused filament fabrication^36^. For this investigation, the experiments were designed using taguchi method with three different levels for three input factors: layer thickness, printing speed, and printing temperature (it indicates the extruder temperature), which was shown in Table 2. Also, two output parameters: Tensile strength and Flexural strength were considered to examine the influence of printing process parameters on the mechanical performance of the printed PLA specimens.

Layer thickness represents the deposition of melted PLA material as a layer with a certain height on the build platform and it was one of the important parameters which enhances the bonding strength of the printed PLA specimens. The printing time and strength of the printed part were influenced by layer thickness. Similarly, improper printing temperature leads to inconsistent and over extrusion with reference to ISO/ASTM 52,900 standard. The printing speed denotes the nozzle velocity in the X/Y directions. So, the un-optimized printing speed may also lead to the gaps in top layers, layer separation and splitting. So, it was highly essential to optimize these parameters to have enhanced mechanical properties with good structural integrity. The experiments consist of three factor and three level, L27 orthogonal array was designed using the Minitab V16 statistical software. The printing was performed one time for each trial such as flexural strength and ultimate tensile strength were used for analysis, as shown in the Table 3.

**Table 3:** Experimental runs for the various combination of input parameters.

Experimental runs	Input parameters			Experimental runs	Input parameters		
	Layer thickness (mm)	Printing speed (mm/s)	Printing temp (°C)		Layer thickness (mm)	Printing speed (mm/s)	Printing temp (°C)
1	0.1	50	195	15	0.15	60	205
2	0.1	50	200	16	0.15	70	195
3	0.1	50	205	17	0.15	70	200
4	0.1	60	195	18	0.15	70	205
5	0.1	60	200	19	0.2	50	195
6	0.1	60	205	20	0.2	50	200
7	0.1	70	195	21	0.2	50	205
8	0.1	70	200	22	0.2	60	195
9	0.1	70	205	23	0.2	60	200
10	0.15	50	195	24	0.2	60	205
11	0.15	50	200	25	0.2	70	195
12	0.15	50	205	26	0.2	70	200
13	0.15	60	195	27	0.2	70	205
14	0.15	60	200				

## Mechanical testing

**Figure 3:**
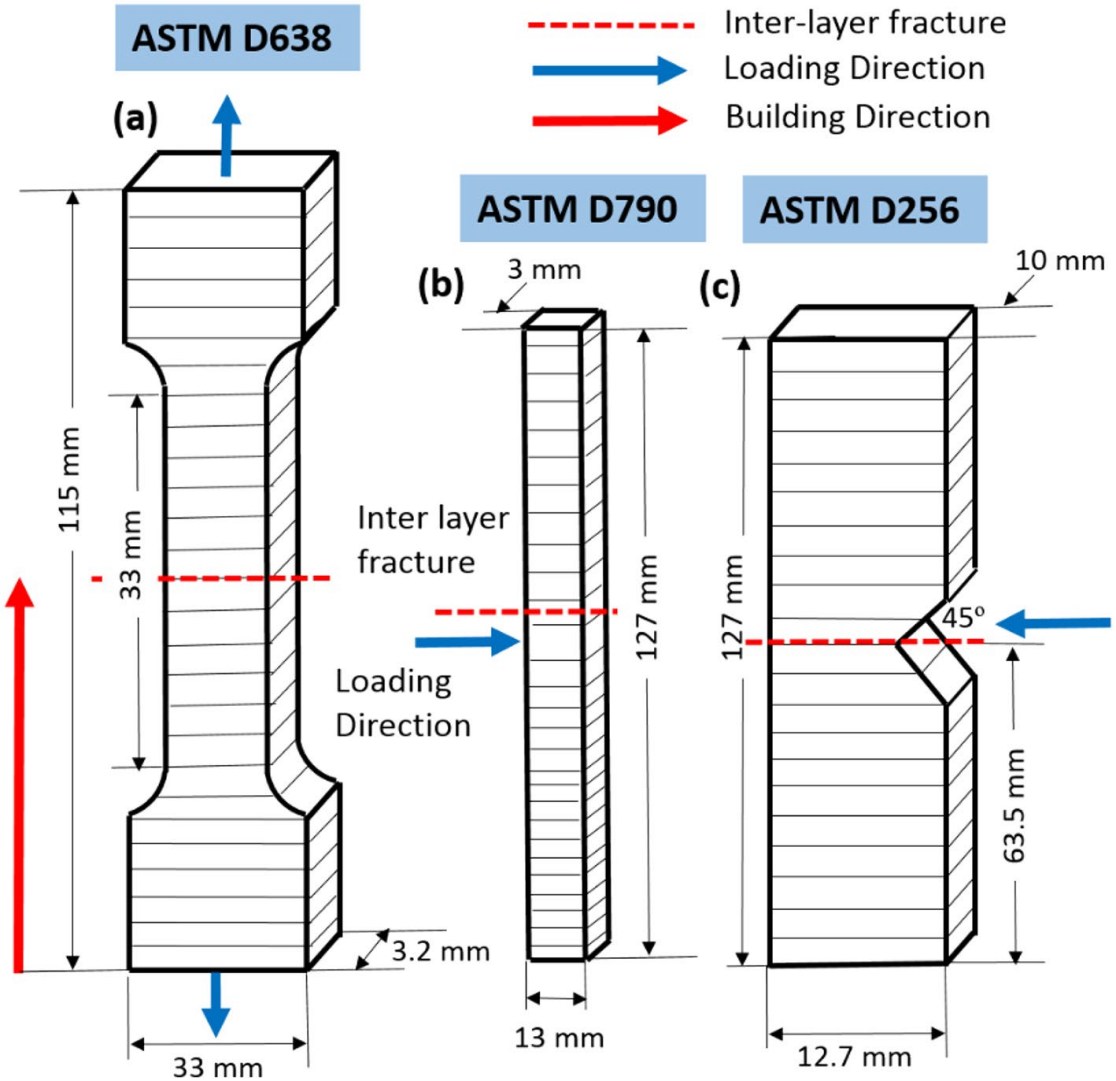
Dimensions and loading conditions of (**a**) tensile, (**b**) Flexural and (**c**) impact specimen.

The mechanical testing was done on the printed specimens based on the ASTM standard. The dimensions of the ASTM standard specimens were shown in the Fig. 2. The Universal Tensile Machine (ATEDigiServoUtm2000) was used to test the ultimate tensile strength of the 3D printed materials. The ASTM D638 Standards were used to create the tensile test specimen (Fig. 2). The flexural strength of the printed specimen was determined using a flexural testing instrument (Eqvimech) based on a 5% deflection of the printed specimen. The ASTM D790 standards were used to create the flexural strength specimen (Fig. 3). Pendulum-type Izod impact apparatus (TFIT-300 N & EN) was used for the evaluation of the impact strength of the printed specimen. ASTM D256 standards were used to create the flexural specimens. The fractured specimens from the mechanical testing were further examined for the fracture studies with the help of Scanning Electron Microscope (Genesis/Veritas Series) analysis and the mode of failure was compared and investigated between the parameters.

## Results and discussion

## Parametric analysis

### Parametric control analysis

The analysis of variance helps in determining the significance of input variables on the response and characterizes the influences of inter- and intra-parametric settings. Analysis of variance (ANOVA) was used to establish the relationship between the input factors and output responses using quadratic terms (linear, square, and two-way interaction), as shown in Table 3. Further, it provides an adjusted sum of squares, probability '*p*' values, R-square and adjusted R-Square values^37^. These values indicate the input factor's significance and contribution to the output responses. The adjusted sum of squares values estimated the percentage contribution of inputs factors and R-squares values. It is noted that the '*p*' values less than 0.05 with R square value of 98.1% and 99.7% confidence level of significantly influence the flexural strength and ultimate tensile strength.

From the Table 4, it can be observed *P* value is less than 0.05 that implies each and individual parameter has significant influence over tensile and flexural strength. Contribution of control factors are calculated based on sequential sum of square, as illustrated in Table3. Layer thickness has major contribution in governing the mechanical properties of printed materials with 90.8 and 92.8%. Printing speed has second major contribution of 5.3 and 4.4% for tensile and flexural strength. Printing temperature has least contribution of 2.2 and 2.5% respectively.

**Table 4:** Analysis of variance (ANOVA) and percentage contribution of input factors on output responses.

Output Response	Source	DF	Seq SS	Adj SS	Adj MS	F	*P*	Contribution %
Flexural strength	Layer thickness	2	1971.73	1971.73	985.86	834	0.000	90.86
	Printing speed	2	116.96	116.96	58.48	49.47	0.000	5.39
	Printing temperature	2	57.73	57.73	28.86	24.42	0.000	2.66
	Error	20	23.64	23.64	1.18			
	Total	26	2170.05					
	R-square : 98.91%							
Tensile strength	Layer thickness	2	892.12	892.12	446.06	4163.26	0.000	92.85
	Printing speed	2	42.36	42.36	21.18	197.66	0.000	4.41
	Printing temperature	2	24.17	24.17	12.09	112.82	0.000	2.52
	Error	20	2.14	2.14	0.11			
	Total	26	960.79					
	R-square : 99.71%							

### Empirical modelling of material extrusion (MEX) process

The regression equations were derived for two output responses (Flexural strength and Ultimate tensile strength) and the three input factors (Layer thickness (L_T_); Printing speed (S_P_); and Printing temperature (T_P_)). The prediction of flexural strength and ultimate tensile strength using the regression Eqs. (1) and (2) expressed as a quadratic form to depict the non-linear relationship of input factors. The empirical model helps in predicting the output responses through input factors^38^. Moreover, the model competence was ensured by the R-square and adjusted R-square values^39^. Here, both values are greater than 92% in both output responses.

1 $${\text{Ultimate Tensile strength }}\left( {{\text{MPa}}} \right) \, = { 81}.{1459 } - { 14}0.{8}*{\text{L}}_{{\rm T}} - \, 0.0{684444}*{\text{S}}_{{\text{P}}} - \, 0.{1}00{333}*{\text{T}}_{{\text{P}}}$$

2 $${\text{Flexural strength}}\left( {{\text{MPa}}} \right) = 130.747 - 209.167*{\text{L}}_{{\text{T}}} - 0.115722*{\text{S}}_{{\text{P}}} - 0.137111*{\text{T}}_{{\text{P}}}$$

**Figure 4:**
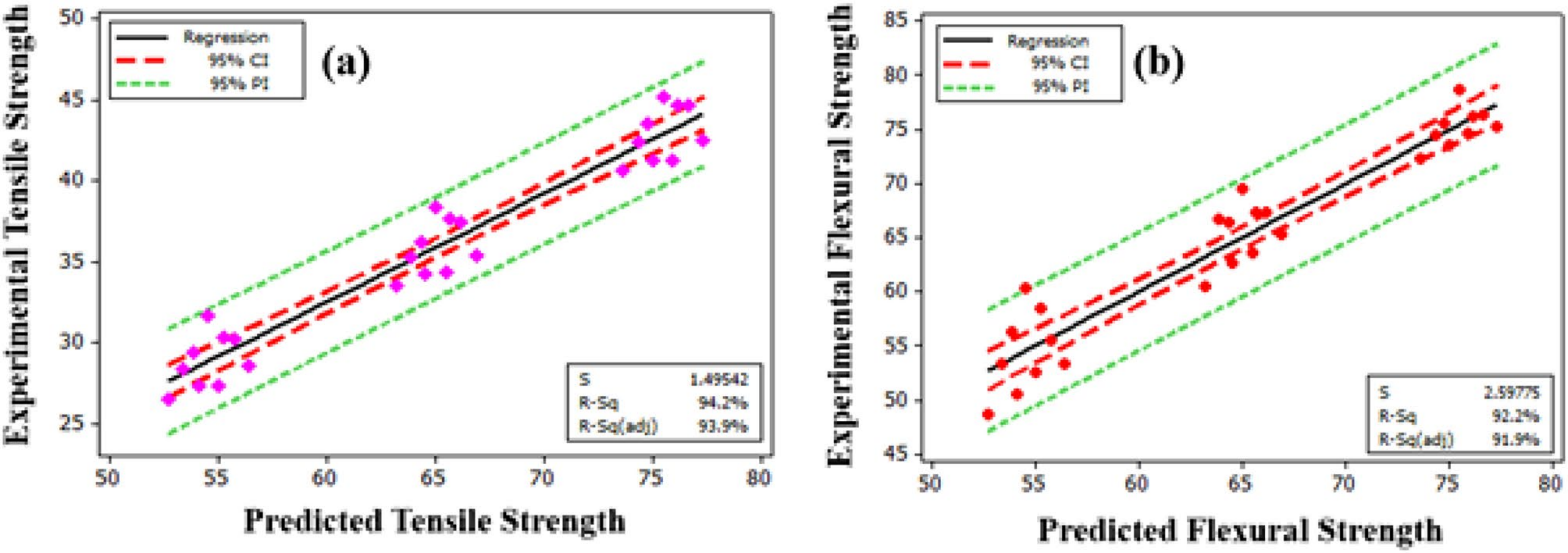
Fitted line plot between predicted and actual values (**a**) Tensile strength and (**b**) ultimate Flexural strength.

The fitted line plot for both output responses (flexural strength and ultimate tensile strength) indicate the correlation between the predicted data from the regression equations and the actual data from the experimental runs, as shown in Fig. 4. It suggests that the actual values agree with the predicted values, which implies the adequacy and reliability of the regression model.

The model which corresponds to the experimental data in the case of flexural strength show R^2^ = 92.2% and for ultimate tensile strength around R^2^ = 94.2%. The high R^2^ value shows the closeness of the predicted results with the model which attributes the supremacy of the empirical model of the process in selected range.

### CRITIC embedded WASPAS based multi objective optimization

Multi-criteria decision making (MCDM) approach assist to solve the decision-making problems where two are more responses are involved. In manufacturing process various MCDM techniques such as Techniques for Order Preferences by Similarity to Ideal Solution (TOPSIS), Grey Relational Analysis (GRA), Principal Component Analysis (PCA) etc. are adopted by expert and materials scientist to attain unique optimal solution for multi response problems. The main drawback in MCDM approach is the difficulties in proper allocation of weightage for each response viz. weightage are allocated with equal value for individual response. To resolve these issues, hybrid MCDM approach such as PCA weighted GRA, Entropy weighted GRA etc., are adopted for assigning weightage for each response based on their criteria. In this research Criteria Importance Through Intercriteria Correlation (CRITIC) embedded Weighted Aggregated Sum Product Assessment (WASPAS)^40,41^ approach is adopted to optimize the process parameters such as Layer thickness (mm), Printing speed (mm/s), Printing temperature (°C). Herein CRITIC approach is adopted to calculate the weightage of individual responses namely tensile and flexural strength of the printed components. This method bases the weights on how the choice problem's contrast intensity and conflict are evaluated. Additionally, this approach does not require human participation throughout the evaluation phase, which helps to automate the decision-making process. Further the calculated weights of each response will be assigned for WASPAS approach. The steps involved in CRITIC embedded WASPAS techniques is follows.

- *Step 1*: It involves formulation of decision matrix with values of response parameter
  3 $${\text{C}} = \, \left[ {{\text{cij}}} \right]_{{{\text{n}}*{\text{m}}}} = \left[ {\begin{array}{*{20}c} {c11 c12 \ldots .c1m} \\ {c21 c22 \ldots .c2m} \\ {. . .} \\ {cn1 cn2 \ldots .cnm} \\ \end{array} } \right]$$
  where cij is performance data of jth response of ith design.
- *Step 2*: Deals with normalization of formulated decision matrix from Eq. (3) the attained values are tabulated in Table 5
  4 $$c_{ij}^{ + } = \frac{{cij - {\text{min}}\left( {cij} \right)}}{{\max \left( {cij} \right) - {\text{min}}\left( {cij} \right)}}$$
- *Step 3*: Determination of correlation coefficient by Eq. (5)
  5 $${\text{CCj}} = \sigma_{j} X \mathop \sum \limits_{j* = 1}^{n} \left( {1 - rjj*} \right)$$
- *Step 4*: objective weights calculation for normalized response
  6 $${\text{OWj}} = \frac{ccj}{{\mathop \sum \nolimits_{j = 1}^{n} ccj}}$$

**Table 5:** Normalized decision matrix obtained in CRITIC method.

Run	Normalization FS	Normalization TS
1	0.8868	0.8571
2	0.9232	0.9686
3	0.8665	0.7894
4	0.9199	0.9696
5	1.0000	1.0000
6	0.8962	0.9120
7	0.8308	0.7884
8	0.8628	0.8491
9	0.7897	0.7548
10	0.5527	0.4733
11	0.6228	0.5858
12	0.4967	0.4216
13	0.6228	0.5981
14	0.6969	0.6338
15	0.5895	0.5203
16	0.4663	0.4147
17	0.6041	0.4696
18	0.3929	0.3774
19	0.1549	0.1103
20	0.2260	0.2010
21	0.1285	0.0469
22	0.3261	0.2031
23	0.3892	0.2761
24	0.2517	0.1535
25	0.0631	0.0426
26	0.1532	0.1007
27	0.0000	0.0000

The calculated OWJ value from Eq. (6) is used in weightage calculation in WASPAS approach. Herein the correlation coefficient and weightages are depicted in Table 6.

**Table 6:** Correlation coefficient and calculated weightage.

	FS	TS	CCj	OWj
FS	1.0000	0.9910	0.0027	0.4848
TS	0.9910	1.0000	0.0029	0.5152

The computed values of output response are used as weightage values in WASPAS method. The steps involved in WASPAS are illustrated as follows.

- *Step 1***:** The output responses are arranged in a form of decision matrix followed by the segregation of beneficial and non-beneficial. In general, the larger, better standards are termed as beneficial criteria. In this research, tensile and flexural strength of the printed components are considered as output response. Higher tensile and flexural strength are needed for the developed components hence it is segregated under beneficial criteria and the normalized decision matrix can be obtain by using Eq. (7) as illustrated in Table 7.
  7 $${\text{Yij }} = \frac{yij}{{\max yij}}$$
- *Step 2***:** It includes calculation of two total relative important variable namely weighted summed model (Qs) and weighted product model Qp. Herrin the weightage calculated in CRITIC approach viz. OWj is used for determining the Qs and Qp values are depicted in Eqs. (8) and (9).
  8 $${\text{Qs}} = \mathop \sum \limits_{j = 1}^{n} Yij*owj$$
  9 $${\text{Qp}} = \mathop \prod \limits_{j = 1}^{n} Yij_{ij}^{OWj}$$
- *Step 3*: Determination of generalized criteria Qc. The Qc values are calculated from Eq. (10) followed by ranking of obtained Qc value. The trail with highest Qc values is considered as the optimal parameter to attain components with higher mechanical strength. Table 8 shows the generalized criteria and optimal solution.
  10 $${\text{Qc}} = \lambda {\text{Qs}} + \left( {{1} - \lambda } \right){\text{Qp}}$$

**Table 7:** Weighted normalized values in WASPAS method.

Normalized FS	Normalized TS	Weighted Normalized FS	Weighted Normalized TS
0.9569	0.9407	0.4639	0.4847
0.9708	0.9870	0.4706	0.5085
0.9491	0.9126	0.4602	0.4702
0.9695	0.9874	0.4700	0.5087
1.0000	1.0000	0.4848	0.5152
0.9605	0.9635	0.4656	0.4964
0.9355	0.9122	0.4536	0.4700
0.9477	0.9374	0.4595	0.4829
0.9199	0.8983	0.4460	0.4628
0.8296	0.7815	0.4022	0.4026
0.8563	0.8282	0.4152	0.4267
0.8082	0.7601	0.3918	0.3916
0.8563	0.8333	0.4152	0.4293
0.8845	0.8481	0.4288	0.4369
0.8436	0.8010	0.4090	0.4126
0.7967	0.7572	0.3862	0.3901
0.8492	0.7800	0.4117	0.4018
0.7687	0.7417	0.3727	0.3821
0.6780	0.6309	0.3287	0.3250
0.7051	0.6685	0.3418	0.3444
0.6680	0.6046	0.3238	0.3115
0.7433	0.6694	0.3603	0.3449
0.7673	0.6997	0.3720	0.3605
0.7149	0.6488	0.3466	0.3343
0.6431	0.6028	0.3118	0.3106
0.6774	0.6269	0.3284	0.3230
0.6190	0.5851	0.3001	0.3015

**Table 8:** Generalized criteria and optimal solution.

Qs	Qp	Qc	Ranking
0.9486	0.9485	0.9486	5
0.9791	0.9791	0.9791	2
0.9303	0.9302	0.9302	7
0.9787	0.9787	0.9787	3
1.0000	1.0000	1.0000	1
0.9620	0.9620	0.9620	4
0.9235	0.9234	0.9235	8
0.9424	0.9424	0.9424	6
0.9088	0.9087	0.9087	9
0.8048	0.8045	0.8046	15
0.8418	0.8417	0.8418	12
0.7834	0.7830	0.7832	16
0.8444	0.8444	0.8444	11
0.8658	0.8656	0.8657	10
0.8216	0.8214	0.8215	13
0.7763	0.7761	0.7762	17
0.8135	0.8128	0.8132	14
0.7548	0.7547	0.7547	18
0.6538	0.6533	0.6535	23
0.6863	0.6860	0.6861	21
0.6353	0.6345	0.6349	25
0.7052	0.7042	0.7047	20
0.7325	0.7317	0.7321	19
0.6809	0.6801	0.6805	22
0.6223	0.6220	0.6222	26
0.6514	0.6509	0.6512	24
0.6016	0.6013	0.6014	27

Herein the values of λ varies from 0 to 1, in this research value of λ is consider as 0.5. The accuracy of λ values can be determined by sensitivity analysis.

Based on ranking it can be depicted lower layer thickness with medium printing speed and medium printing speed delivers optimal mechanical properties.

## Contour plot-based optimization

### Flexural strength

**Figure 5:**
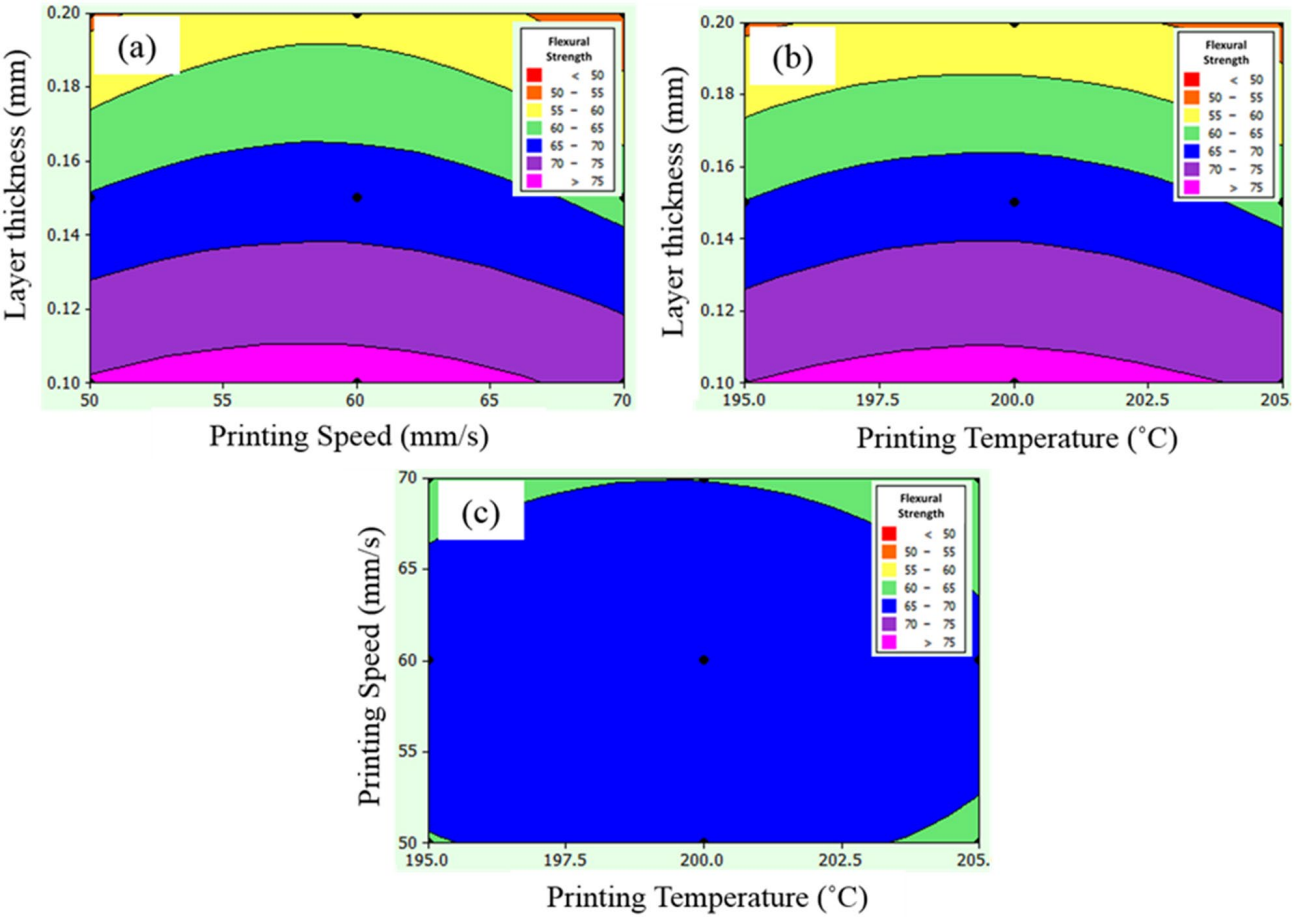
Contour plot for flexural strength (**a**) printing speed and layer thickness, (**b**) printing temperature and layer thickness, and (**c**) printing temperature and printing speed.

The 2D contour plot, as shown in Fig. 5a–c respectively represent the effect of input factors such as layer thickness, printing speed and printing temperature on the flexural strength and ultimate tensile strength, respectively. Flexural strength is the ability of the 3D printed PLA polymer to withstand bending stress which ensures the adhesion of the printed layers^42^. It is an intensive property that can be influenced by process input factors, not by the dimension of the specimen.

The contour plot of flexural strength was obtained based on input factors' hold values, such as layer thickness of 0.10 mm, printing speed of 60 mm/s, and printing temperature of 200 °C. Figure 5a represents maximum flexural strength greater than 75 MPa at minimum layer thickness (0.1 mm) for printing speeds ranging from 50 to 65 mm/s. Similarly, Fig. 5b shows maximum flexural strength (> 75 MPa) at minimum layer thickness (0.10 mm) for all printing temperatures (195–205 °C). However, the moderate flexural strength around 60–65 MPa was obtained at a maximum printing speed of 70 mm/s with a minimum printing temperature of 195 °C and vice-versa (50 mm/s of printing speed and 205 °C of printing temperature), as shown in Fig. 5c.

### Ultimate tensile strength

**Figure 6:**
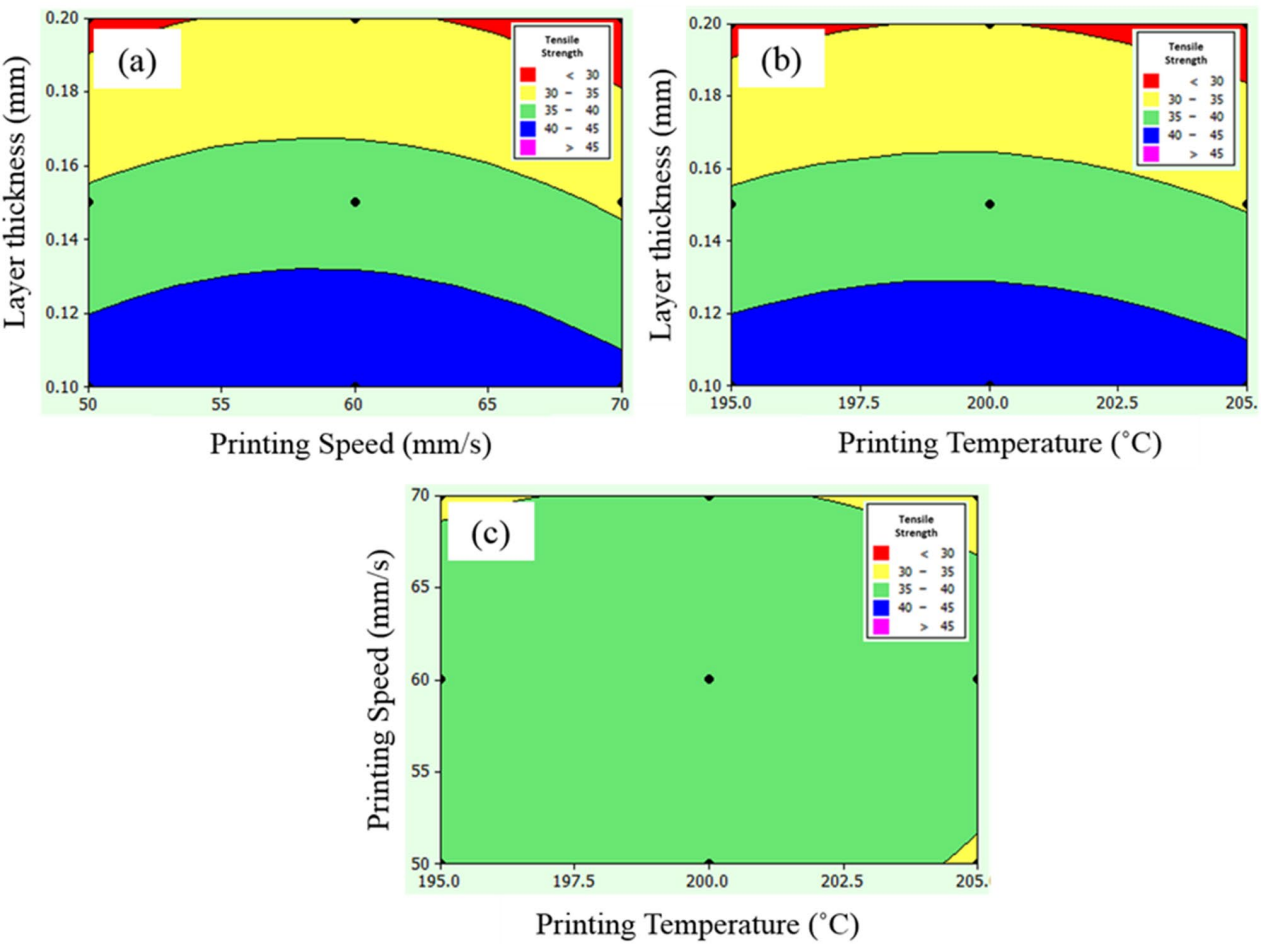
Contour plot for ultimate tensile strength (**a**) printing speed and layer thickness, (**b**) printing temperature and layer thickness, and (**c**) printing temperature and printing speed.

The ultimate tensile strength is the maximum load the PLA cross-section carries at the tensile condition^43^. There is a maximum ultimate tensile strength (> 45 MPa) with minimum layer thickness with moderate printing speed (55–60 mm/s) and printing temperature (197.5–200 °C), as shown in Fig. 6a and b, respectively. Mainly, the tensile strength is significantly affected by print speed and layer thickness. The fundamental reason behind the print speed is that the layer increment of PLA material unbalances the process which result in compromised mechanical attributes. The air-gap in the layers and the stiffness mainly drives the tensile strength in poly-lectic acid^28^.

The balanced control over the air-gap reduction and improvement in the stiffness which is directly linked with print temperature and speed, and layer thickness enhances the tensile strength. Rajpurohit and Dave^44^ resulted in improved tensile strength through combined control over layer thickness and speed. This control provides maximum area for bonding and the number of printed layers (such as 60 mm/s) enhances the overall stiffness^23^. Similarly, Zandi et al.^45^ resulted in significant influence of print speed on the tensile strength because the process parameter significantly controls the increment in printed layers and their number. Moreover, the printing temperature and speed combinedly reduces airgap at optimized process parameters using 0.150 mm layer thickness. The ultimate tensile strength was reduced from 45 to 35 MPa as printing speed (70 mm/s), and printing temperature (205 °C) increased to their corresponding maximum limits (Fig. 6c).

## Fractured surface analysis

**Figure 7:**
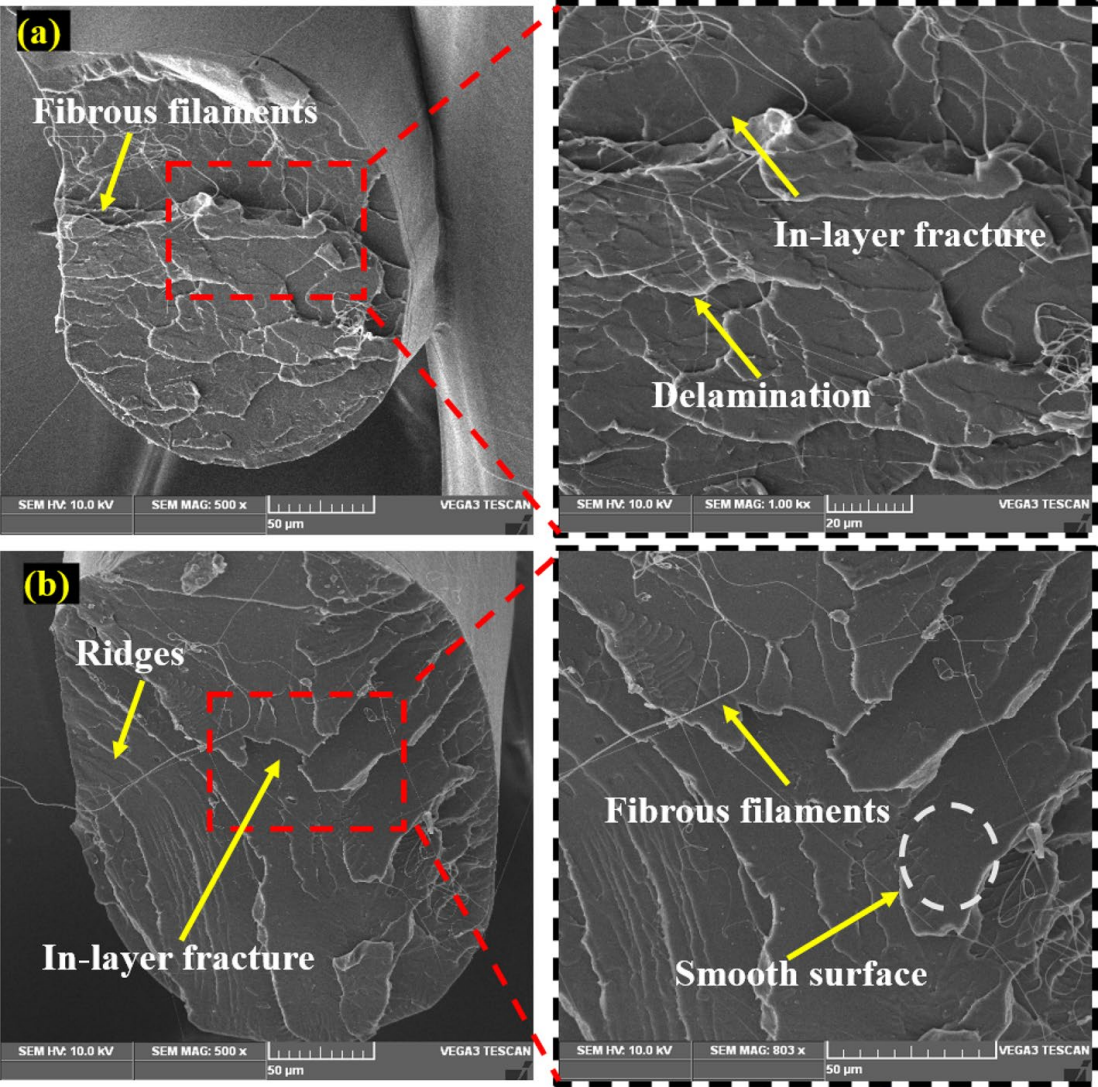
Microstructure of the tensile specimens (**a**–**b**) fracture surface under optimum input parameters.

**Figure 8:**
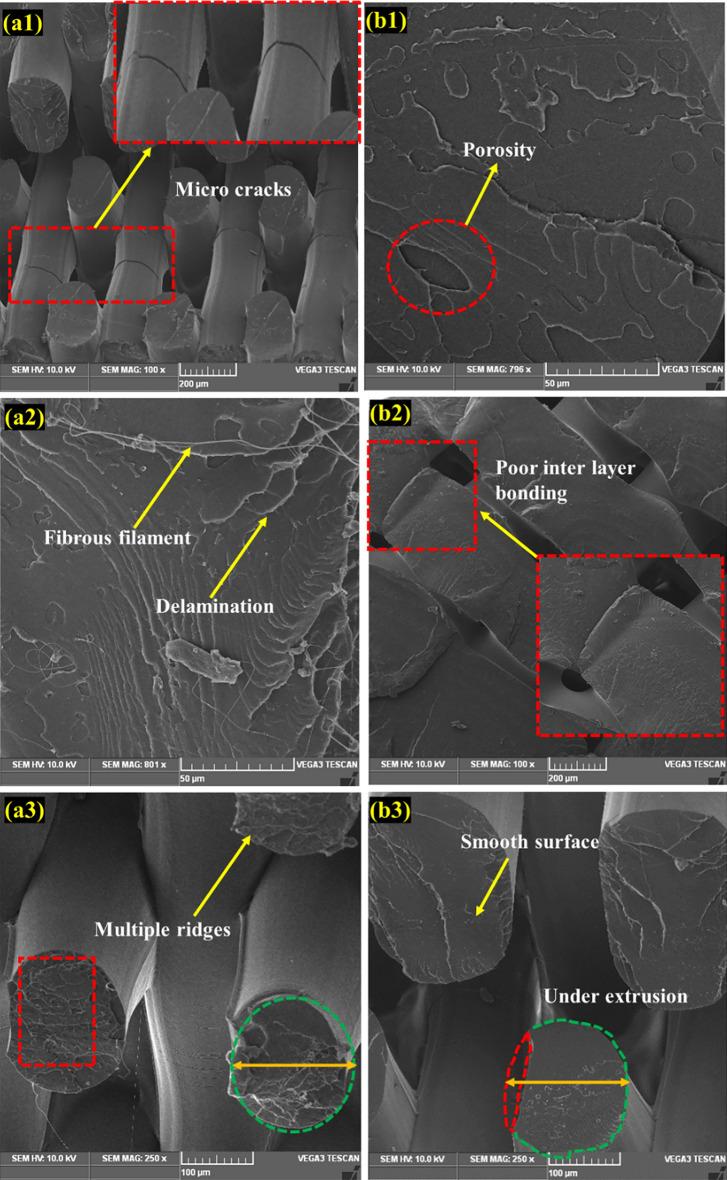
Comparison of fracture mechanism of tensile specimens (**a**1–**a**3) optimum parameters (Layer thickness = 0.1 mm, Printing speed = 58 mm/s, Printing temperature = 199 °C), (**b**1–**b**3) non-optimum parameters (Layer thickness = 0.2 mm, Printing speed = 70 mm/s, Printing temperature = 195 °C).

**Figure 9:**
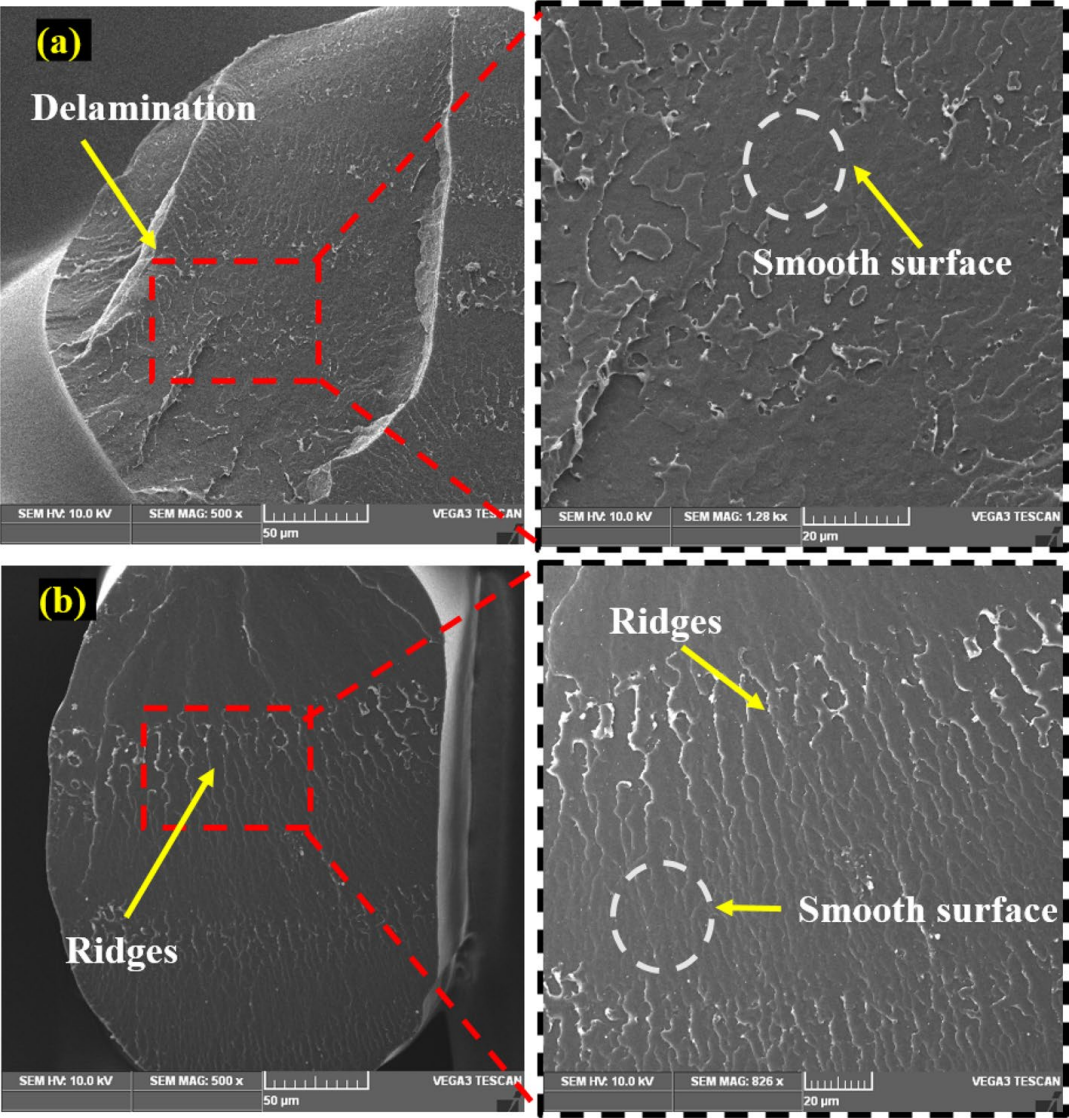
Microstructural analysis of the flexural specimens (**a**-**b**) fracture surface under optimum input parameters.

Figure 7a and b show a scanning electron microscopy (SEM) image of the fractured surface of the PLA tensile specimen under MEX-optimized conditions. The MEX process parameters had a significant effect on the tensile properties of the fabricated parts. The layer thickness exhibits a linear correlation with the specimen's tensile strength. It was revealed that increasing the layer thickness reduces the tensile strength of the specimen. The bonding strength was increased by fusing multiple layers with reduced thickness, and fused layers of small thickness achieved the closely packed interlayers. Fibrous filaments with more delamination were seen on the broken surface which signifies the higher bonding strength and also these was an absence of voids and porosity, these mechanisms were observed in Fig. 8a2. Zhang et al. suggested that the ridged fractured surface represents the effective layer deposition had occurred^46^. The clear circular shaped extrusion without any defects was observed in the Fig. 8a3 which was due to the optimum printing speed of 58 mm/s. However, some microcracks were observed in the fractured surface and that depicts the stress concentrated regions (Fig. 9a1). On comparison of optimum (Layer thickness = 0.1 mm, Printing speed = 58 mm/s, Printing temperature = 199 °C) and un-optimized parameters (Layer thickness = 0.2 mm, Printing speed = 70 mm/s, Printing temperature = 195 °C) which was shown in Fig. 8, The voids and poor interlayer bonding was evident on the fractured surface of the Specimen printed with un-optimized parameters which was shown in the Fig. 8b1, b2. Similarly, under extrusion was also observed in the un-optimized print setting. The under extrusion was shown in the Fig. 8b3. These defects had a considerable effect on the strength of the printed material, resulting in inferior specimens^47^. The excision of these defects enhances the mechanical properties of the specimens. This reveals that 0.1 mm layer thickness with 58 mm/s printing speed at 199 °C printing temperature yields smooth layers with good bonding. The fibrous filaments from the fractured surface depict the ductility of the PLA. Inter-layer cracks were visible on the broken surface, revealing the strong bonding layers. Furthermore, this fracture analysis also substantiates the Taguchi—CRITIC embedded WASPAS approach based optimum conditions.

Furthermore, the printing temperature and speed influence the tensile strength of the specimens. The high-speed printing (70 mm/s) was attributed to the extruded material's volume shrinking. High-speed printing inhibits the continuous flow of materials and causes discounters in layer thickness. It also contributes to the formation of porosity and poor interlayer bonding. These defects were shown in the Fig. 8b1–b3. This challenge was overcome by maintaining a constant printing speed of 58 mm/s and an appropriate printing temperature of 199 °C, allowing good fusion and solidification time for the extruded materials, thereby improving printing accuracy and contributing to the enhancement of the tensile strength.

The fractured surface of the printed flexural specimen was examined under optimal process parameters, as illustrated in Fig. 9a and b. The SEM analysis of the fractured surface of the flexural specimen revealed a smooth and ridged surface with no pore formation. Similarly, there was a delamination and absence of micro-cracks, which contributed to the delayed flexural failure. These cracked surfaces demonstrate that the printing speed of 58 mm/s, a layer thickness of 0.1 mm, and printing temperature of 199 °C substantially enhanced flexural strength.

**Figure 10:**
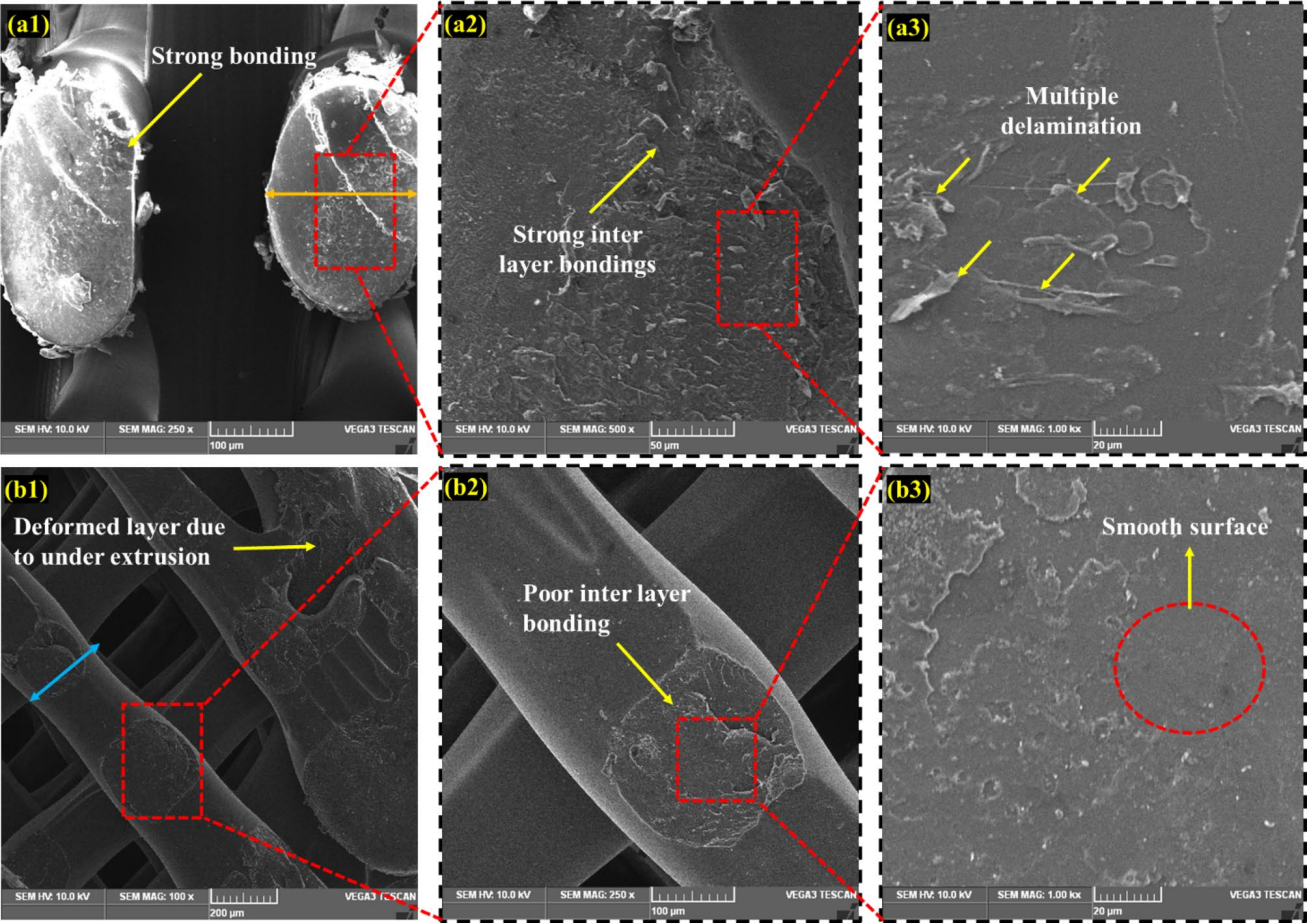
Comparison of fracture mechanism of tensile specimens (**a**1–**a**3) optimum parameters (Layer thickness = 0.1 mm, Printing speed = 58 mm/s, Printing temperature = 199 °C), (**b**1–**b**3) un-optimum parameters (Layer thickness = 0.2 mm, Printing speed = 70 mm/s, Printing temperature = 195 °C).

On the comparison of optimum and un-optimized parameters (Fig. 10), Specimen printed with optimized parameters shows more delamination on the fractured surface, indicating strong interfacial bonding (Fig. 10a1–a3). In the case of specimen printed with un-optimized parameters, the smooth fracture surface from the Fig. 10b2, b3 reveals the poor interlayer bonding and also deformed layer thickness due to under extrusion was noted in the Fig. 10b1 which was due to the higher printing speed of 70 mm/s. From the flexural strength analysis results, it was observed that increasing the layer thickness reduces the flexural strength of the specimens. Because increased layer thickness minimizes the contact area between neighboring layers, resulting in low bonding strength and, as a result, poor resistance to bending effect and flexural strength. The evaluation found that increasing the printing speed and temperature decreases the flexural strength of the specimens. Because higher printing speed causes porosity and fracture formation, resulting in poor bending strength^48^. Similarly, high printing temperature affects the structural stability of the filaments by overheating the filaments, whereas lower printing temperature does not create sufficient wettability between each layer. As a result, the optimized input parameters overcame the limitations.

**Figure 11:**
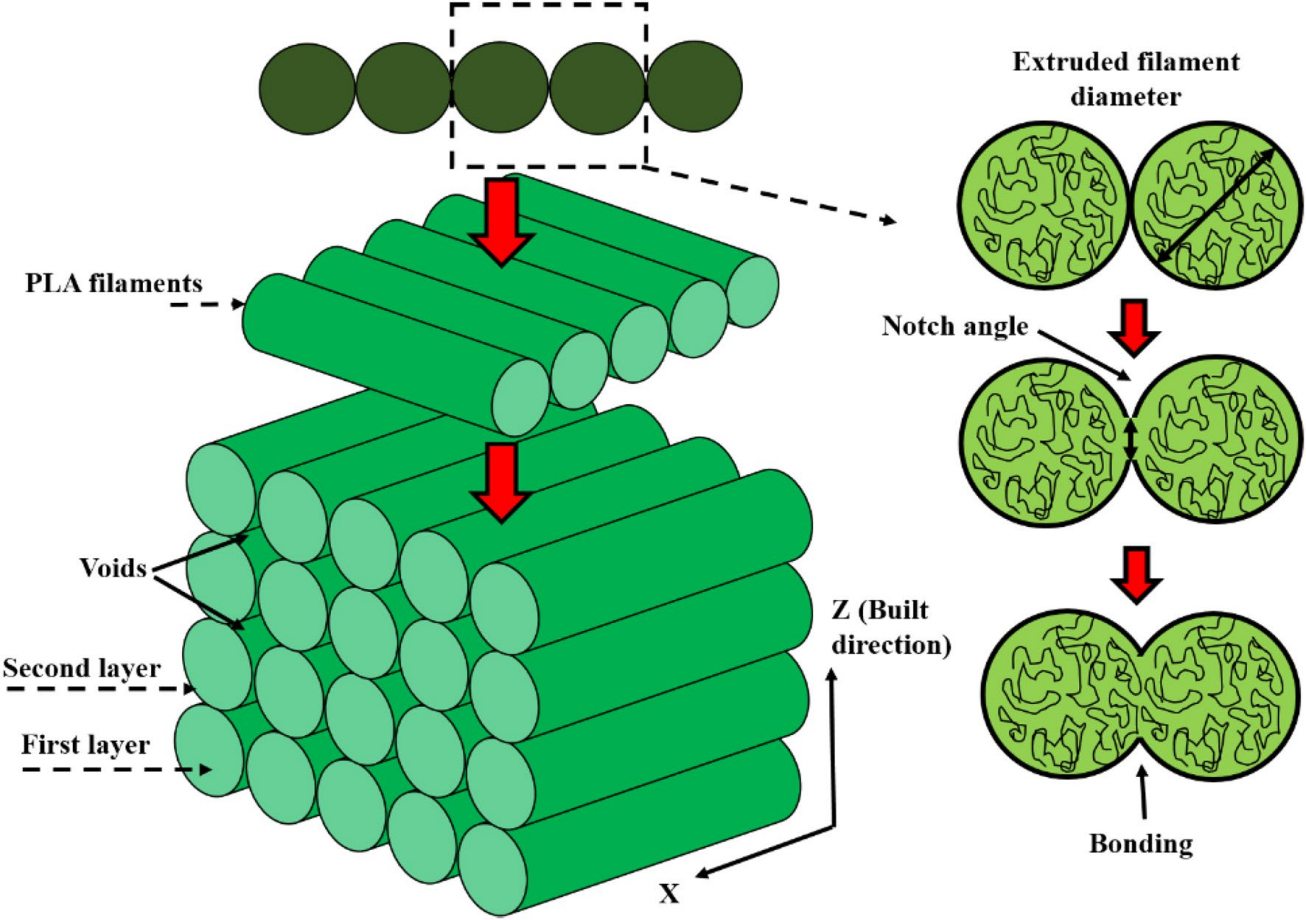
Schematic representation of bonding mechanism.

**Figure 12:**
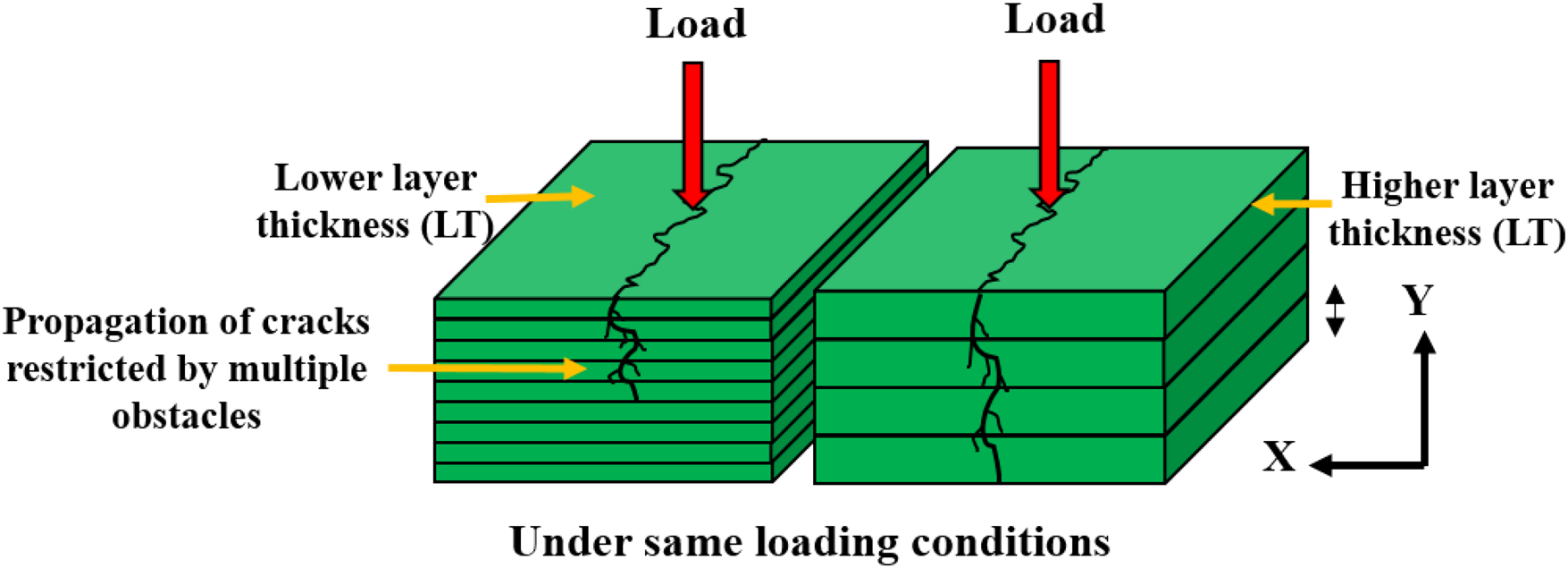
Schematic representation on influence of layer thickness on crack propagation.

Figure 11 depicts the mechanism of interfacial bonding between the layers. This densely packed structure avoids pores and confined spaces between the layers. Moreover, the closely packed interlayers resulting from the smaller layer thickness substantially impeded crack growth. This mechanism was shown in the schematic representation in Fig. 12. Each printed layer inhibits the propagation of cracks. As a result, parts built with the maximum number of layers and the minimum thickness significantly resist crack propagation. Whenever a specimen was printed with a relatively high layer thickness, voids with poor interlayer bonding appear (Fig. 8b1, b2), and that region acts as the stress concentrated zone when the load is applied.

## Impact analysis

**Figure 13:**
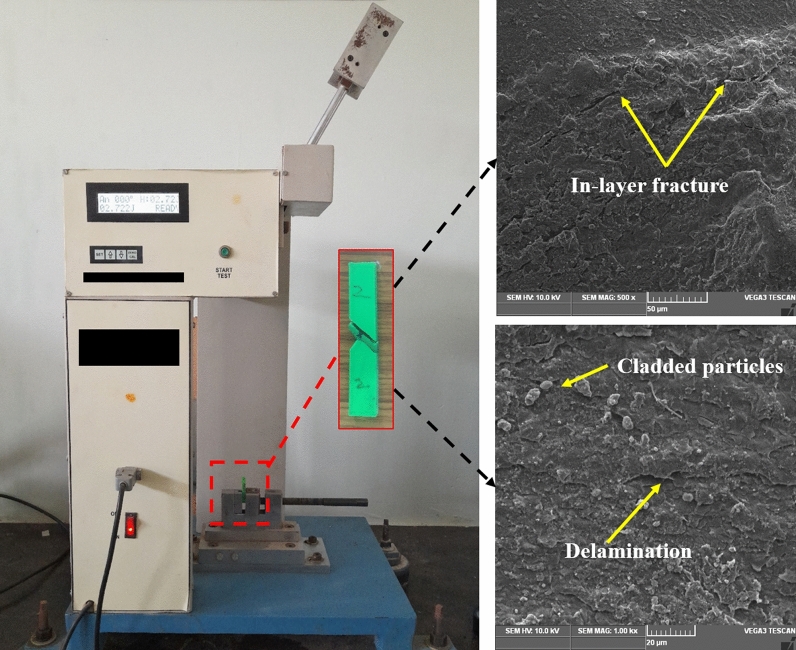
Printed PLA (Izod) impact specimen under optimum input parameters and fractured surface.

The impact (Izod) specimen was also built using the optimum criteria for evaluating impact strength. The defect such as porosity, poor adhesion, and molecular bonding had the most significant influence on enhancing impact strength. Cohesiveness and the structural morphology of the molecules are two intrinsic material attributes that substantially affect impact strength. Compared to the previous findings^49^, the maximum impact strength of 6.21 kJ/m^2^ was achieved with the optimal process parameters, indicating a 17.16% increase. This reveals that reduced layer thickness, proper printing speed, and appropriate printing temperature improve the impact strength of printed parts. Multiple fused layers with reference to ISO/ASTM 52,900 standard restricted the propagation of cracks with minimum thickness due to enhanced diffusion between the layers. The printing speed of 58 mm/s minimizes defects such as voids and porosity by building strong interfacial adhesion between the layers and enabling sufficient fusion time. Because these imperfections impaired the capability of the printed parts to absorb impact energy. The printed PLA was devoid of defects, as evidenced by SEM images of the impact fracture surface, as shown in Fig. 13.

## Conclusion

The flexural strength and ultimate tensile strength of MEX processed PLA was investigated using Taguchi—CRITIC embedded WASPAS approach. The Taguchi method was adopted for experimental planning and to understand the effect of process parameter over mechanical properties of printed PLA parts. CRITIC embedded WASPAS method was used to attain optimal unique solution of parameter to achieve optimal mechanical strength viz. flexural and tensile strength. The results are summarized as follows.

- From the analysis of variance, it has been observed that P values are less than 0.05 for all the process parameter. Hence each and individual parameter has significance over output response and layer thickness play vital role in governing the material strength.
- Increased printing speed and temperature decreased the flexural strength of the specimens which was due to the lack of sufficient time for the bonding of interlayers and also evident that the higher printing speed causes porosity and fracture formation, resulting in poor bending strength.
- Through impact analysis, it was recognized that multiple fused layers by 0.1 mm layer thickness restrict the propagation of cracks. In which each layer with minimum layer thickness act as an obstacle to the propagation of cracks. Similarly, the printing speed of 58 mm/s minimizes defects such as voids and porosity by building strong interfacial adhesion between the layers and enabling sufficient fusion time.
- From the Taguchi—CRITIC embedded WASPAS approach, optimal printing parameters were identified as layer thickness of 0.1 mm with printing speed of 60 mm/s and 200 °C printing temperature which results in ultimate tensile strength of 45.22 MPa, flexural strength of 78.64 MPa and impact strength of 6.21 kJ/m^2^. These results were substantiated by displaying good interlayer bonding on the fractured surface under different loading conditions.

## Data Availability

The datasets used and analyzed during the current study are available from the corresponding author on reasonable request.
